# Artificial Intelligence in Radiology: Advancing Precision, Accuracy, and Early Detection in Cancer Diagnosis

**DOI:** 10.7759/cureus.100102

**Published:** 2025-12-26

**Authors:** Pragati Gurjar, Saad Khan Mayana, Sravan Krishna Reddy Annadevula, Bhanupriya Singh, Kumar Sambhav, Sapana B. Shah

**Affiliations:** 1 Department of Health, Indian Institute of Health Management Research (IIHMR) University, Jaipur, Jaipur, IND; 2 Department of Radiology, Narayana Medical College, Nellore, IND; 3 Department of Radiodiagnosis, Sanjay Gandhi Postgraduate Institute of Medical Sciences, Lucknow, IND; 4 Department of Anatomy, All India Institute of Medical Sciences, Bilaspur, Bilaspur, IND; 5 Department of Anatomy, Dr. N.D. Desai Faculty of Medical Science and Research, Dharmsinh Desai University, Nadiad, IND

**Keywords:** artificial intelligence, cancer diagnosis, clinical decision support systems, precision oncology, radiology

## Abstract

Artificial intelligence (AI) is rapidly transforming oncologic radiology, enabling earlier detection, greater precision, and more personalized care. Yet much of the literature remains fragmented into disease-specific studies or narrow performance assessments. This review addresses that gap through a narrative thematic synthesis of research published between 2019 and 2025, identified from major biomedical and engineering databases and selected for clinical relevance, translational value, and policy significance. Unlike prior reviews that catalog isolated applications, it organizes evidence into cross-cutting frameworks that redefine radiology’s role in cancer care. These include advances in precision imaging and early detection, the integration of multimodal data for richer disease characterization, and the use of AI in prognosis and treatment monitoring. Equally, the review highlights challenges of model explainability, federated learning, equity, and workforce adaptation as determinants of adoption. By situating these themes within Clinical Decision Support Systems (CDSS) and broader healthcare infrastructures, the analysis shows that AI’s significance lies less in isolated accuracy gains than in its transparency, inclusivity, and adaptability across contexts. The review concludes that the decisive priority now is to build global collaborations, robust validation, and ethical frameworks that ensure AI evolves as an inclusive ecosystem capable of delivering equitable improvements in cancer care worldwide.

## Introduction and background

Cancer is one of the most daunting global health problems, causing millions of new cases each year and challenging healthcare services. Radiology plays a central role in oncology, providing invaluable tools of detection, staging, treatment planning, and monitoring [1]. The introduction of multimodal imaging, computed tomography (CT), magnetic resonance imaging (MRI), positron emission tomography (PET), ultrasound, and hybrid technologies has increased the precision of the diagnosis but also generated massive amounts of complex data [2]. Radiologists are supposed to interpret these data under time pressure, and this causes deviations, fatigue, and delays in reporting. In low-resource settings, the lack of qualified professionals also contributes to the existing differences in timely diagnosis and access to treatment [3].

Artificial intelligence (AI) has emerged as a potential solution because it can automate parts of the interpretation process, improve accuracy, and identify subtle imaging patterns that humans may overlook [4]. Modern AI systems often use convolutional neural networks, which learn layered or “hierarchical” features by identifying simple patterns first and progressively combining them into more complex structures [5]. More recent advances include transformer-based models, which are designed to capture long-range relationships within imaging data and enhance tasks such as lesion detection and segmentation [6]. These systems can support not only detection but also risk stratification, prognosis, and treatment monitoring, all of which are central to precision oncology [7]. In certain applications, for example, in AI-assisted mammography for breast cancer screening and lung nodule classification on low-dose CT, large externally validated studies have reported performance comparable to that of expert radiologists, though such outcomes remain highly context-dependent and cannot be generalized across all cancers or modalities [8]. Although AI has shown promising results in specific applications, its broader clinical impact remains limited by issues of generalizability, workflow integration, regulatory uncertainty, and the need for substantial human oversight.

**Figure 1:**
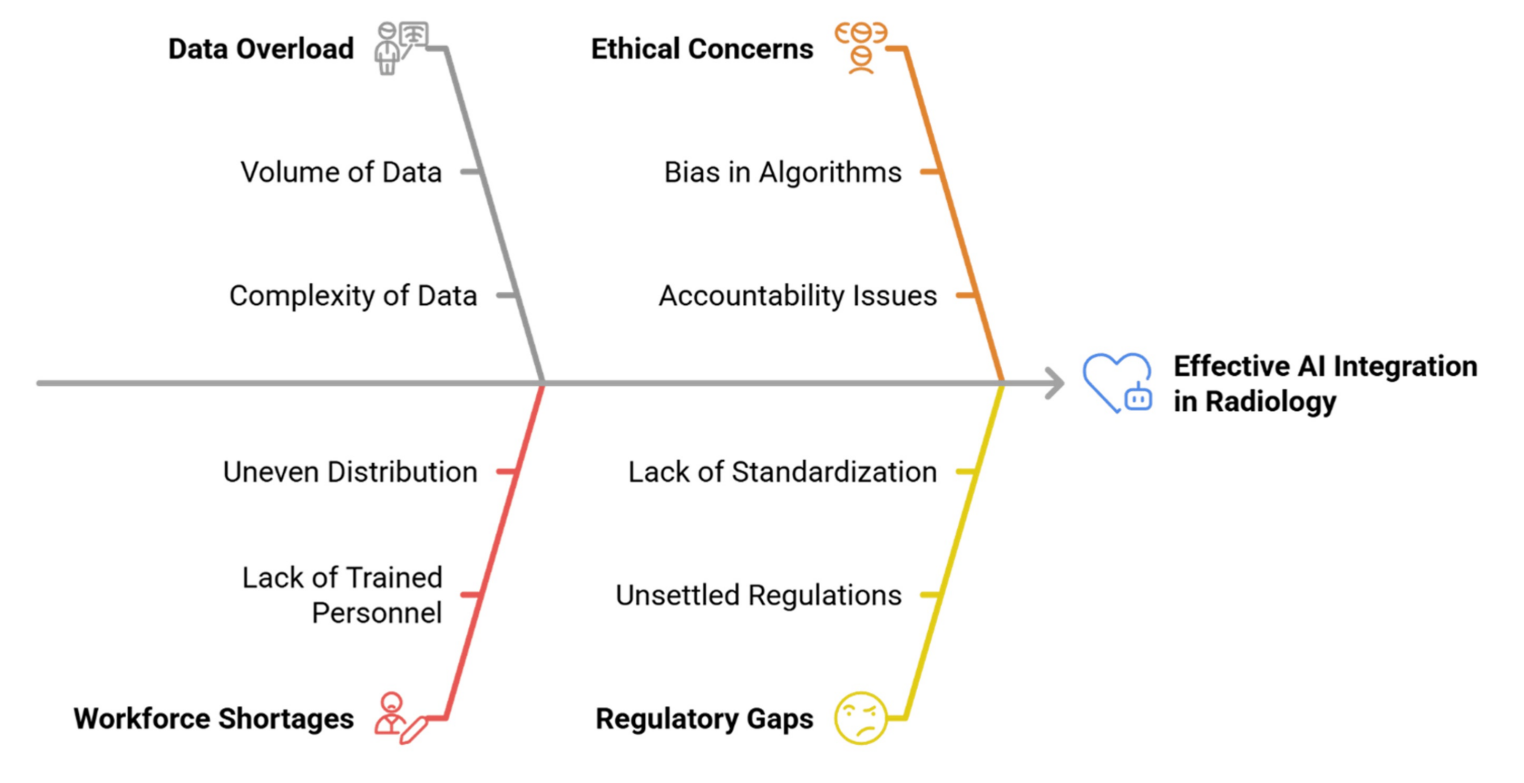
Cross-cutting themes of artificial intelligence in oncologic radiology Image Credit: Created by the authors.

Yet progress has been slower than expected. Models that perform well on curated datasets do not necessarily generalize to other populations, scanners, or institutions [9]. Regulatory frameworks remain unsettled, especially for continuously learning systems [10]. Ethical issues are also acute: biased training data, which refers to datasets that underrepresent certain populations, may create additional disparities; black-box predictions, meaning AI outputs whose internal decision processes are not transparent, can compromise clinician trust; and liability in AI-assisted errors remains ambiguous because responsibility is not clearly attributable to either the clinician or the algorithm [11]. The transformative potential of AI is thus bracketed by the existence of important restrictions that require critical analysis [12]. AI intersects with several dimensions of radiology, which form a thematic framework that goes beyond disease-specific applications, as shown in Figure 1.

This review takes a different stance than disease-specific studies that list the applications of AI in breast, lung, or prostate cancer. Although this type of work proves that it is possible, it does not show the whole picture. What is direly needed is conceptual clarity on how AI is transforming radiology as a field. This review is thus organized thematically, discussing AI along cross-cutting dimensions: precision imaging, early detection, multimodality integration, explainability, federated learning, equity, and governance. These themes go beyond individual cancers to discuss more general changes that are transforming clinical practice and the structure of health systems.

Radiology itself is becoming a predictive science, rather than a diagnostic science. Clinicians are increasingly using imaging not only as a means to diagnose disease, but to predict its progression and customize therapy [13]. AI expedites this transition by calculating quantitative biomarkers and matching them with genomic and clinical data and making it feasible to track them through time. This intersection makes radiology the hub of personalized oncology and generates difficult questions: How do clinicians interpret predictions in the absence of transparency? Who is responsible in case the algorithms go wrong? Will these tools reduce or develop cancer disparities all over the globe? Such questions highlight that AI is not only a technological innovation but also a broader societal challenge that influences trust, equity, and responsibility in clinical practice [14].

In this spirit, the review will aid in shedding some light on the connection between AI and oncologic radiology by addressing these concerns. It does not provide a list of applications but rather takes into account the frameworks, possibilities, and obstacles that will determine whether AI will become a safe, equitable, and sustainable component of cancer care or not. Finally, AI should not be considered a replacement for human experience, or a panacea; instead, of coming across as a component that should be integrated carefully in, interdisciplinary manner and monitored.

Objectives of the review

This review will provide an overview of AI in oncologic radiology based on cross-cutting themes and not disease-specific applications. It describes the technological advances that can be used to provide accurate images, early diagnosis, and a multimodal approach, and evaluates how these approaches have affected clinical outcomes in terms of diagnostic reproducibility, prognostic accuracy, and treatment monitoring. It also deals with systemic problems, including workflow redesign, regulatory control, adaptation of the workforce, fairness, transparency, and ethical accountability issues of ethics. Special attention is given to explainable AI as a means of fostering trust, federated learning as a strategy to overcome data silos without compromising privacy, and equity-based solutions as pathways to extend advanced diagnostics to low-resource settings. Finally, this review clarifies what AI can achieve today, where evidence remains limited, and what infrastructural, collaborative, and policy measures are required to ensure responsible global adoption of AI in cancer care.

## Review

Methodological considerations

The current review uses a narrative, thematic synthesis of literature published between January 2019 and January 2025, covering both technical advances and early clinical applications of AI in radiology. Relevant studies were identified through structured searches in PubMed, Scopus, IEEE Xplore, and arXiv using combinations of keywords such as AI, oncology, radiology, radiomics, deep learning, and multimodal imaging. Studies were included when they presented original research, systematic reviews, or conceptual analyses directly relevant to oncologic imaging, clinical workflow integration, or regulatory and ethical considerations. To maintain a focus on clinically meaningful applications, studies were excluded when they lacked clinical relevance or provided insufficient methodological detail to support interpretation within a healthcare context. While this review does not adopt a formal systematic review methodology, the literature included spans applications across CT, MRI, PET, ultrasound, and multimodal AI systems, offering a broad qualitative representation of ongoing developments. The narrative design was chosen to synthesize cross-cutting themes rather than quantify frequencies of specific modalities or study designs. This approach, while interdisciplinary and comprehensive, may underrepresent studies conducted in low-resource settings or those published in languages other than English, underscoring the need for more globally inclusive evidence in future research.

AI as an enabler of precision radiology

Radiology is moving to biomarker-based and quantitative practice. This transition may be accelerated with the help of AI that detects reproducible imaging characteristics that define tumor heterogeneity and that, in turn, are correlated with clinical outcomes [15,16]. Compared to the traditional interpretation that is exposed to training and fatigue, the AI-based analysis is more regular, particularly in prostate MRI reporting and breast density reporting [17]. In addition to reducing inter-observer variability, they also enable multi-center research in which similar measures are required in order to compare them. Equally important is AI’s capacity to transform tumor quantification [18]. Automated segmentation and volumetric measurements offer reproducible measurements of tumor burden that can then be used to track changes longitudinally to provide more accurate staging and monitoring. These volumetric markers may be strong determinants of survival and represent a potential surrogate endpoint in oncology [19]. The significant obstacles remain. Models trained on small or single-site data tend to fail when used in other populations, thus requiring federated and collaborative models that both protect individual privacy and enhance generalizability [20]. In addition, issues of equity need to be considered, as the technology that will enable advanced AI is concentrated in systems with high resources [21]. The need to ensure that the concept of precision radiology is accessible across various contexts will be important to ensure that this concept benefits patients in different parts of the world [22]. Table 1 outlines the key areas in which AI is improving precision radiology, with the conventional shortcomings, clinical implications, and exemplary literature.

**Table 1:** Contributions and challenges of AI in precision radiology

Domain	Traditional Limitation	AI Contribution	Clinical Implication	Representative References
Interpretation	Subjective, variable across readers (training, fatigue)	Extraction of radiomic signatures capturing heterogeneity, morphology, and texture	More objective, biomarker-driven imaging	[23]
Reproducibility	High inter-observer variation (e.g., prostate MRI, breast density)	AI scoring systems align more consistently with pathology-confirmed outcomes	Greater standardization across centers and trials	[15]
Quantification	Manual measurements are prone to error and inconsistency	Automated segmentation and volumetric tumor burden assessment	Reliable metrics for staging, monitoring, and survival correlations	[7]
Generalizability	Models underperform across institutions and populations	Federated learning enables training on distributed datasets while preserving privacy	Broader applicability, improved external validity	[11]
Equity	Benefits concentrated in high-income systems	Potential for portable, low-resource, adaptable solutions	Success depends on accessibility as much as accuracy	[5]

Early detection beyond traditional screening

The key to better survival is early detection of cancer, but existing screening regimens are limited by sensitivity and interpretation inconsistency. AI improves screening by detecting invisible changes that predate radiographically visible lesions and decreasing false positives [24]. Deep learning algorithms trained on large mammography datasets have performed as well or better than experts and have reduced unnecessary recalls. Equivalent outcomes have been realized in lung cancer through the use of low-dose CT to determine the classification of nodules using AI [25]. Beyond improving existing workflows, AI supports personalized screening. Risk scores that are built based on a combination of imaging, demographic, and genomic variables allow stratified surveillance [26]. The high-risk individuals would be subjected to intensive follow-up, and the low-risk groups would not be subjected to unnecessary imaging, and a cost would be reduced along with overdiagnosis [27]. Although promising, such strategies will need to be validated in the future to demonstrate their effect on long-term outcomes.

**Figure 2:**
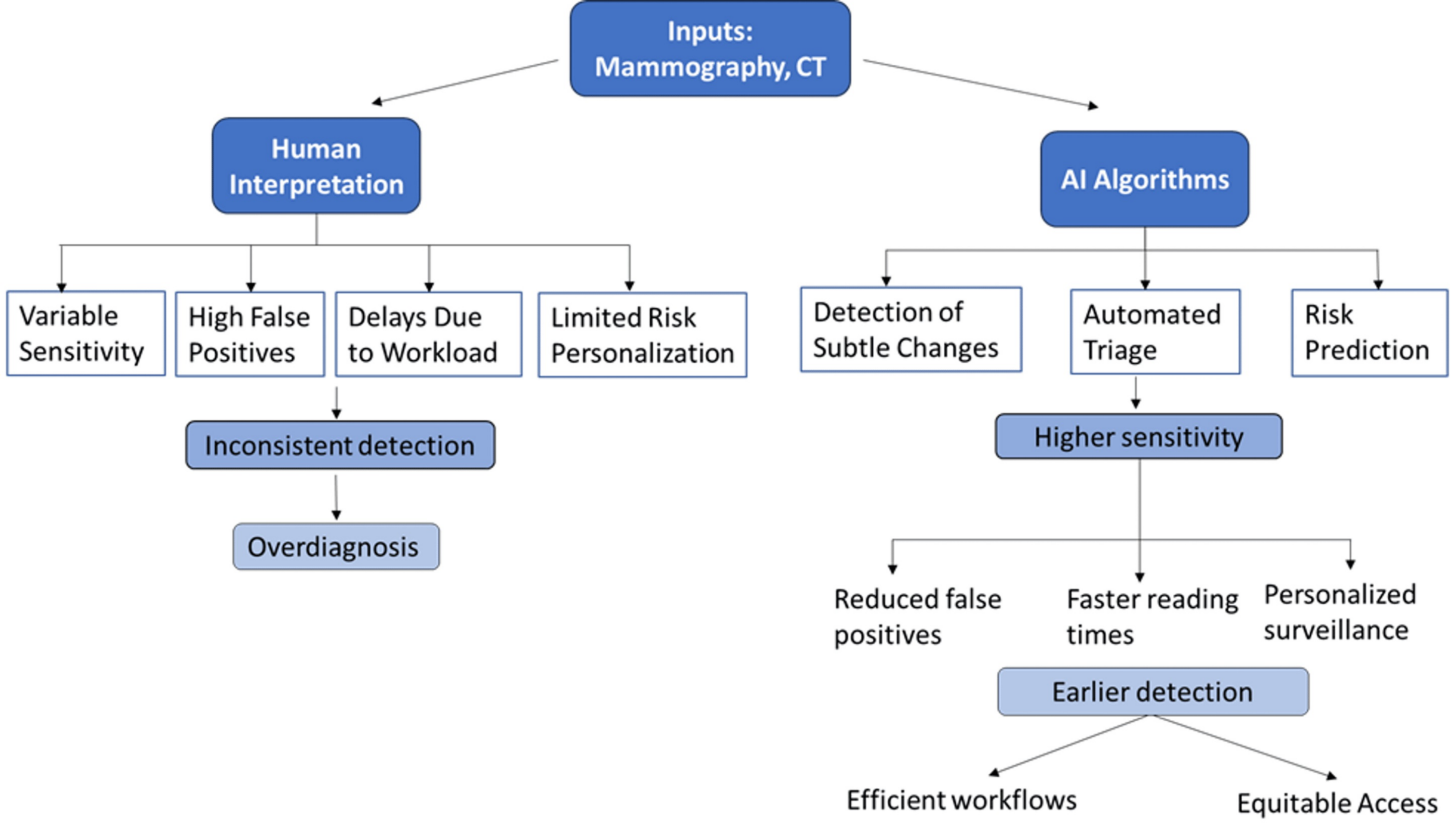
Traditional versus AI-enhanced screening workflows Image Credit: Created by the authors.

AI also acts as a triage tool. In mass screening, the majority of the scans are normal; automated identification of low-risk studies decreases the workload and reading times of radiologists by almost 50% without sacrificing accuracy [28]. Nevertheless, such systems can have poor performance in other populations, where the breast density, smoking rates, and disease patterns are different. Equity concerns are acute. The majority of training data is sourced in high-income areas, and its scope is restricted to low- and middle-income countries (LMICs) populations [29]. Without diverse representation, AI may reinforce disparities. Automation bias is another risk factor, as clinicians can over-rely on algorithm outputs in emergency decisions. Transparency tools like saliency maps and confidence scores are thus necessary to continue to build trust and prevent AI from taking over the role of experts [30]. AI amplifies the capabilities of early detection by making screening more accurate, forecasting risks before disease symptoms appear, and automating processes [31]. Its influence will be defined by how well it is validated, the inclusiveness of the data, and how it is meaningfully incorporated into health systems. Figure 2 shows how AI is used to streamline screening and facilitate risk-based surveillance.

Cross-modality synergies

The different imaging modalities provide different information: CT provides anatomic detail, MRI soft-tissue contrast, PET metabolic activity, and ultrasound vascular dynamics [32]. Historically, these datasets have been assessed separately. AI enables cross-modal integration, which leads to a unified representation that enhances diagnosis and management [10]. Multimodal AI has already shown clinical promise. In neuro-oncology, multiparametric MRI with PET enhances the classification and molecular subtype prediction of gliomas. In lung cancer, combining CT characteristics with PET uptake information helps in better characterization of the nodules and avoids unnecessary biopsies [33]. These two examples demonstrate how AI can reveal cross-domain correlations that cannot be seen by human beings. Treatment planning also benefits. Radiotherapy models that incorporate anatomy via CT, contrast via MRI, and metabolism via PET provide more accurate target delineation and optimization of doses [34]. In systemic therapy, the combination of MRI volumetrics and PET response markers allows adaptive treatment strategies, which are vital to make changes in time.

Harmonization across modalities is equally critical. The inter-center protocol variability compromises the comparability of multi-center data, but AI-based harmonization can standardize the resolution and intensity to pool data across centers to build federated learning. This harmonization is not purely technical; it forms the foundation of the creation of generalizable tools that could be used across institutions. Nevertheless, integration comes with obstacles [35]. Multimodal AI requires a lot of computational resources and quality control. Interpretability is further complicated when models combine heterogeneous data, which is a matter of concern in high-stakes oncology decisions [36]. In addition, multimodal imaging is not as widely available in LMICs, posing a threat to increasing disparities. Remedy of these conditions will require the creation of light clinical models of transparent models that are technically advanced and clinically viable [37]. However, the convergence of AI is likely to bring about a day when cancer imaging will cease to be divided by modality but rather will manifest itself as a set of interconnected and biomarker-abundant disease representations. These developments have the potential to render radiology into predictive precision-based oncology [16].

AI in image reconstruction and enhancement

AI has started to transform the basics of image reconstruction, mitigating the age-old conflict between safety, efficiency, and diagnostic clarity [38]. Trade-offs are the norm in conventional imaging, where lower radiation in CT or shorter MRI sequences always come at the cost of quality, whereas longer sequences or higher doses carry higher risks to the patient [12]. AI-powered reconstruction algorithms are changing this trade-off by producing diagnostically sound images at lower doses and faster rates [39]. Deep learning reconstruction in CT shows that noise suppression can maintain image quality at a much lower exposure value [15]. This translates to patients being subjected to much less radiation without compromising on diagnostic confidence [40]. MRI has also been advantaged by increasing the speed of acquisition, whereby the undersampled data can be reconstructed into high-quality images [17]. This change is particularly useful among susceptible groups, such as children and critically ill patients who cannot withstand extended scan times [41].

The other advantage of AI is that it can eliminate artifacts that interfere with interpretation [9]. Patient motion, irregular breathing, or contrast variations can be reduced by algorithms, which limits the necessity of repeat imaging [19]. Such corrections are cost-saving, avoid exposing patients to extra radiation, and enhance the efficiency of workflow [42]. Also promising is the super-resolution enabled by AI, which can improve the resolution of fine details beyond the capabilities of the hardware, helping detect small or early-stage lesions that would otherwise go unnoticed [13]. Despite these advances, concerns remain. The majority of reconstruction algorithms are trained on a specific scanner model and protocol, and it is not clear how well they will perform in other institutions with different equipment [16]. There is also the danger of hallucinated structures, whereby AI inadvertently introduces or changes features, which can mislead clinical decisions [18]. Future verification in a wide variety of real-world environments is still lacking, and regulators are not keen on approving systems that modify raw imaging data.

Equity is an additional challenge. Cutting-edge reconstruction software requires powerful hardware and computational resources, which are not readily available in resource-poor health systems [10]. Unless there are purposeful plans to accommodate scalability and access to AI-assisted reconstruction, it could increase the image quality gap between low- and high-income contexts [11]. The difference with the review on reconstruction is that instead of focusing on the performance, it focuses on how it affects the strategic aspect of radiology, such as its ability to provide cleaner images, its efficiency in the workflow, and its scale of accessibility to people across the globe [5].

Explainability and trust in AI

The use of AI in radiology has renewed one of the oldest problems in medical technology: how to build trust in systems whose inner workings are often incomprehensible [43]. Although most algorithms are currently operating at expert levels, there have been concerns about the black-box nature of these systems, which clinicians must be held responsible for. Explainability is not an optional add-on to the AI system, but a condition of safe and responsible use [44]. In oncology, where clinical decisions are frequently used to drive biopsies, surgeries, or systemic therapies, interpretability offers a safeguard against automation bias. There is also increasing regulatory pressure to have explainable outputs as a condition of approval, indicating that explainability is part of governance and not purely a technical goal [45].

The pursuit of interpretability presents unresolved dilemmas. There remains a trade-off between interpretability and performance: stronger models are usually less transparent, while simpler, explainable systems may lack accuracy [13]. Most explainability research is still limited to proof-of-concept studies, with little prospective clinical validation. Equity dimensions also complicate adoption, since opaque systems can further erode trust in populations already underserved by healthcare. Ensuring that AI outputs are explainable across diverse contexts is therefore as important as achieving technical performance [2]. Table 2 summarizes common methodological approaches to explainability, outlining their functions, limitations, and implications for clinical and ethical practice.

**Table 2:** Approaches, limitations, and implications of explainability in AI for radiology Grad-CAM: gradient-weighted Class Activation Mapping; SHAP: SHapley Additive exPlanations; LIME: Local Interpretable Model-agnostic Explanations

Approach/Issue	Function	Limitations	Clinical/Ethical Implications	References
Saliency maps/Grad-CAM	Highlight regions of the image most influential to predictions	Often unstable, may emphasize irrelevant regions	Provide visual cues for radiologists; support acceptance	[12]
Feature attribution (e.g., SHAP, LIME)	Quantify the contribution of individual features	Difficult to apply to complex imaging data	Allow interrogation of AI reasoning; align outputs with knowledge	[29]
Counterfactual explanations	Show how small input changes alter predictions	Still largely experimental in radiology	Potential to improve clinician understanding and patient trust	[3]
Transparency in regulation	Require interpretable outputs for approval	Standards are inconsistent across regions	Enhances accountability and safety	[24]
Equity considerations	Promote trust across diverse populations	Opaque systems may deepen mistrust in underserved settings	Explainability is essential for global adoption and fairness	[19]

Federated learning and data democratization

In radiology, AI requires large, heterogeneous datasets, but data availability is one of the most significant barriers to the field [46]. Regulations on privacy, institutional silos, and unequal global representation limit the development of resilient models [12]. Federated learning has been proposed as a solution, enabling several institutions to jointly train algorithms without exchanging raw patient data [47]. In this decentralized model, models are trained locally, and only parameters are shared, while confidentiality is maintained [8]. The outcome has been the ability to develop systems that mirror the diversity of global populations and imaging practices [48]. Current evidence suggests that federated models match or even outperform centralized models in terms of performance and improve generalizability [19].

The democratizing potential is profound. By allowing institutions in high- and low-resource areas to participate, federated learning can make AI more representative of the general population than the limited datasets of a few well-financed hubs [49]. This inclusivity is essential in reducing bias, particularly in oncology, where the distribution of disease patterns and imaging availability is highly varied [22].

Still, significant hurdles exist. Federated learning demands the standardization of data, safe infrastructure, and computational resources at all participating sites [50]. The quality of annotation or imaging protocols can vary and may negatively impact model robustness [7]. The level of governance is poor, and intellectual property, accountability, and liability are yet to be resolved [24]. Besides, despite the minimized barriers to participation, non-digitized institutions are locked out, which continues inequalities [29]. The distinctiveness of this review is that it frames federated learning not merely as a technical workaround but as a pathway to equity: a collaborative, global effort that can only succeed if underrepresented institutions are deliberately included [11].

Equity and global health perspectives

AI in radiology is frequently presented as a universal solution, but its advantages are not distributed equally [21]. In high-resource health systems, AI is applied to optimize processes, enhance screening regimes, and treatment planning [32]. By contrast, in LMICs, radiology infrastructure may be inadequate, and there may be a severe shortage of trained specialists [19]. The issue is not whether AI can enhance imaging performance but whether it can bridge the diagnostic gaps in the areas where needs are most significant [27]. AI can help fill gaps in expertise in underserved areas. Ultrasound has lightweight diagnostic tools that can inform the acquisition of images, generate initial interpretations, and offer point-of-care assistance to non-specialists using AI [38]. Mobile technologies, used in rural clinics, bring cancer detection to the community where radiologists are not available [17]. AI triage can also be added to teleradiology networks, where urgent cases are flagged locally and more complex scans are sent to distant experts.

Despite this promise, barriers remain substantial. The majority of AI models are trained and tested on North American, European, and East Asian populations, whose genetic, demographic, and disease characteristics are not comparable with those in Africa, South Asia, or Latin America [25]. Models trained on these datasets could perform poorly in deployment in the rest of the world, inducing a systematic bias into clinical practice [34]. Another constraint is infrastructure: consistent electricity, connectivity, and digital storage are requirements of AI use, but are unreliable in resource-constrained environments. Affordability is equally critical. Proprietary AI systems are prohibitively expensive, which further promotes the use of external vendors and contributes to inequalities [37]. The lack of open-source options or cost-sharing schemes can make it available only to those hospitals that have the funds [20]. To realize the global potential of AI, models need to be made to operate on low-power devices, tested on diverse populations, and supported by the training of local healthcare providers [30]. Contrary to many reviews, which define equity as a secondary issue, this discussion takes it as the main standard of AI utility [36]. The innovation is in re-conceptualizing equity not as something that is incidental to technological advancement, but as the variable that can make the difference between AI shrinking or widening the global divide in cancer care. Table 3 outlines the major barriers and solutions for equitable AI adoption in radiology.

**Table 3:** Equity challenges and strategies for global AI adoption in radiology LMICs: low- and middle-income countries

Challenge	Impact on Care	Potential Solutions	References
Dataset bias	Reduced accuracy when applied to underrepresented populations (Africa, South Asia, Latin America)	Build globally representative datasets; incentivize multi-regional collaborations; encourage open data sharing.	[35]
Infrastructure gaps	Limited deployment due to unreliable electricity, connectivity, and storage	Develop lightweight AI tools; design offline-compatible platforms; invest in basic digital infrastructure.	[27]
Affordability	Proprietary systems create dependence on external vendors and exclude low-resource settings.	Open-source models; cost-sharing frameworks; public-private partnerships	[6]
Workforce shortages	Lack of trained radiologists in LMICs leads to delays and reduced diagnostic capacity.	AI-enabled ultrasound and mobile diagnostics; targeted training for local providers	[42]
Trust and adoption	Opaque AI risks mistrust, especially in underserved populations	Transparent models; culturally sensitive deployment; community engagement	[14]

Integration with Clinical Decision Support Systems (CDSS)

AI is frequently described as a diagnostic tool, yet its actual transformative power will be found when it is incorporated into more comprehensive CDSS [49]. Radiology data is usually not sufficient to base treatment planning on; combining imaging data with pathology, genomics, clinical records, and patient history is necessary [28]. The CDSS platforms that are AI-enabled can combine these aspects and move radiology out of the realm of a solitary interpretive exercise and into the center of multidisciplinary care. Within these systems, AI can provide more than detection [50]. An example is a lung nodule found on CT automatically connected to pathology results, genomic markers, and treatment guidelines recorded in the electronic health record [35]. Rather than providing a single output, the system places the finding in the context of a continuum of diagnostic and therapeutic information. This holistic strategy minimizes care fragmentation, aids evidence-based decision-making, and makes it easier to follow guidelines [22].

AI dashboards that integrate imaging and molecular and clinical information could support multidisciplinary tumor boards and their siloed backgrounds of expertise [31]. These systems make decision-making more standardized, cut down on cognitive load, and speed up consensus. At the population level, the CDSS platform that collects imaging and clinical data can be used to feed cancer registries and quality improvement initiatives, where AI can be used to influence the population [37]. Integration is not without challenges. Interoperability gaps, data silos, and inconsistent standards continue to slow the exchange of imaging systems with electronic health records [24]. Privacy issues become more pronounced when sensitive genomic, imaging, and clinical data are combined in one platform. Resistance to perceived encroachment on autonomy may be another issue that clinicians face when AI outputs are offered as prescriptive rather than advisory [30]. Unlike many previous reviews, which are limited to the accuracy of a diagnostic measure, integration is highlighted in this review as the most accurate measure of impact [21]. The novelty of this review lies in demonstrating that AI attains real clinical significance only when integrated into decision support systems that unify imaging, molecular, and clinical data to drive precision oncology [33].

AI for prognosis and treatment monitoring

Radiology has been appreciated for its diagnostic ability, but AI is taking it further into prognosis and monitoring of continuous treatment [51]. Using features in imaging that humans cannot see, AI tools can forecast disease progression, risk-stratify patients, and identify early biomarkers of therapeutic response [12]. This makes imaging a dynamic account of cancer evolution rather than a static snapshot [25]. Baseline scan-trained prognostic models can detect subtle predictors of aggressiveness, allowing clinicians to adjust therapy intensity [31]. Patients expected to have indolent disease can be spared the toxicity of unnecessary therapies, whereas high-risk patients can be prioritized for more aggressive interventions [19]. This categorization is of special importance because oncology is becoming more precision-based, meaning that it needs more refined prediction than a categorization [35].

Another frontier is treatment monitoring. The conventional response assessment is mostly size-specific and does not provide any biological alterations at the initial stages available [21]. The AI algorithms can recognize metabolic or textural alterations at the point in time when shrinkage has not begun yet, giving an early indication of success in the treatment process [2]. Such abilities minimize the time spent in changing ineffective regimens and improve the survival outcomes [28]. The immunotherapy proves to be promising and complicated at the same time. Most criteria commonly include pseudoprogression as a failure in treatment, resulting in an early termination [33]. AI models that combine temporal data of imaging with systemic characteristics enhance the distinction between actual progression and those that are immune-related, thus maintaining patients on effective treatments [18]. In addition to immunotherapy, AI can be useful during radiotherapy, where it can forecast response and toxicity, and in chemotherapy, where it can predict systemic adverse events, including organ damage or muscle loss [37].

**Figure 3:**
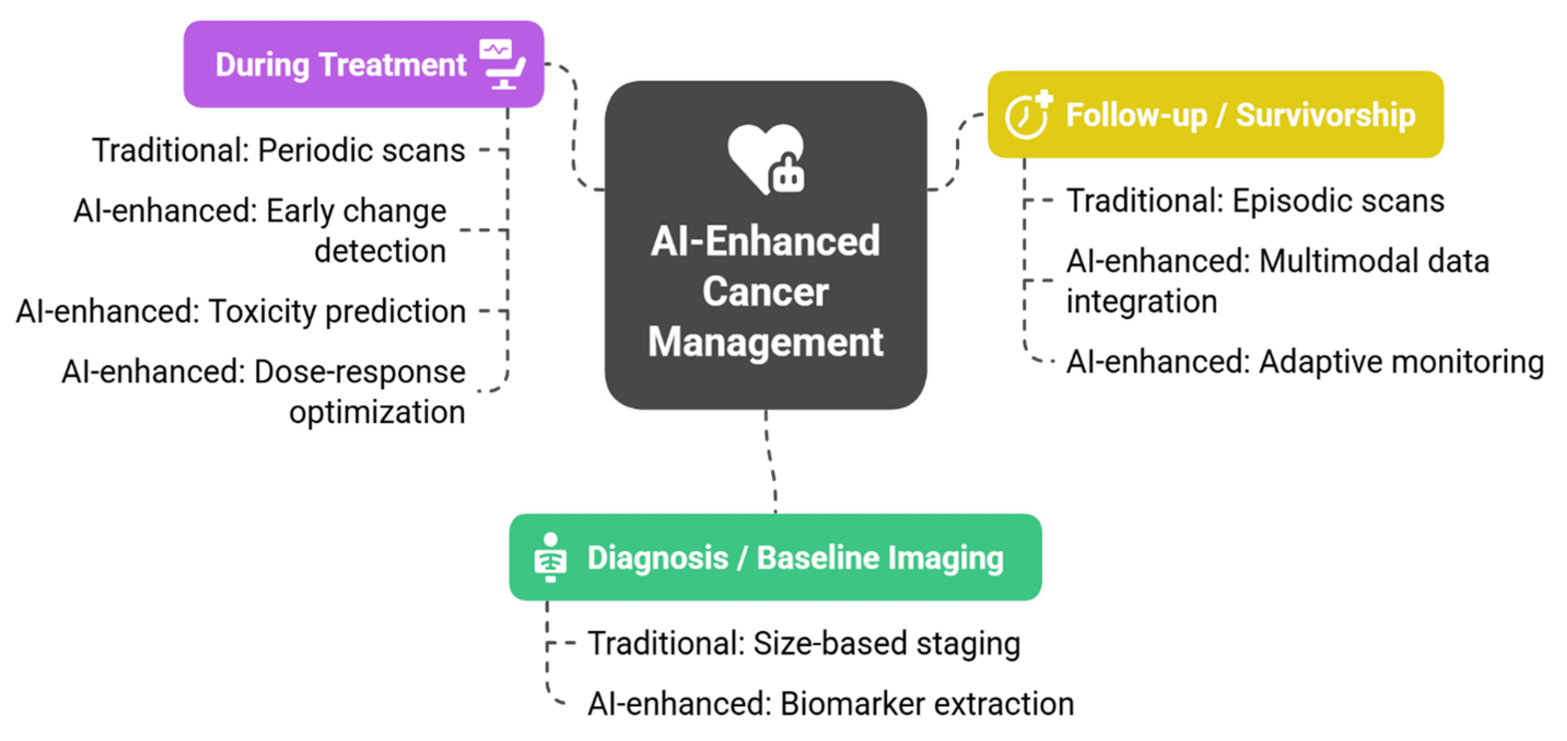
AI-enhanced cancer management across diagnosis, treatment, and follow-up Image Credit: Created by the authors.

Some obstacles include the requirement of prospective validation of the tests in other tumor types, imaging protocol standardization, and a demonstration that the prognostic information has a significant impact on the clinical outcomes [9]. Ethical issues are also urgent; predictive models can be abused in terms of rationing care or insurance in the case of non-regulation [14]. The novelty in this is that prognosis and monitoring are redefined as a transformation of radiology itself. Unlike reviews that view AI as a modest enhancement, this perspective emphasizes how imaging is being reshaped into a longitudinal rather than episodic tool for disease management across therapies [23]. AI thus expands radiology beyond initial diagnosis to dynamic, continuous care, enabling earlier response evaluation and adaptive follow-up, as illustrated in Figure 3.

Ethical, legal, and workforce transformations

It is not only a technical but also an ethical, legal, and professional change to adopt AI in radiology [27]. These dimensions not only indicate the possibility of the implementation of AI but also the trust in it and its maintenance [41]. Accountability is a central concern. In the case of AI-assisted interpretation, it is hard to attribute errors to anybody [36]. Radiologists will still be legally responsible, but their judgment can be influenced by black-box algorithms [12]. Regulators are struggling to audit and approve systems that evolve, and there is a debate about liability in the age of models that learn continuously [48]. Bias adds another layer of complexity. Algorithms trained on unrepresentative datasets are likely to contribute to inequities, as certain groups of people are systematically underserved [22]. Ethical adoption must involve intentional inclusion of diverse data and disclosure of shortcomings [30]. Informed consent is also changing: patients have a right to understand when AI is involved in their care, the role it serves, and its limitations [17].

The workforce implications are equally significant. Instead of replacing radiologists, AI will probably transform their role [43]. Radiologists can evolve into auditors of AI results, multimodal data integrators, and consultants who combine machine predictions with clinical knowledge [15]. To succeed in this capacity, training must incorporate data literacy, ethics, and interdisciplinary collaboration [25]. Professional resistance should not be underestimated. Fears of deskilling, job replacement, and loss of control are genuine [38]. Addressing them requires evidence that AI can strengthen professional authority rather than diminish it [29]. Such a transition will also require open communication and institutional support to build confidence. In contrast to most other reviews, which subordinate ethics and workforce concerns, this discussion foregrounds them as determinants of AI adoption. The originality of this work is in showing that regulation, accountability, and professional adaptation are not afterthoughts but prerequisites for making AI safe, equitable, and sustainable in oncology.

Limitations and future considerations

There are still obstacles on the way to the practical implementation of AI in radiology. Most systems are built on highly curated datasets that do not reflect real-world variability, leading to poor generalizability across populations, scanners, and clinical workflows. A related concern is overfitting, where models perform exceptionally well on training data but fail to maintain accuracy when deployed in diverse clinical environments. Most validation remains retrospective, and long-term, prospective trials are still rare. In addition, explainability continues to lag behind performance, making clinicians reluctant to rely on opaque outputs for high-stakes decisions. Security vulnerabilities further complicate deployment; AI imaging models can be susceptible to adversarial attacks, in which small input perturbations intentionally designed to be imperceptible to humans can mislead an algorithm’s prediction. Another emerging challenge relates to continuously learning systems that adapt after deployment and may not fit within existing regulatory frameworks designed for static technologies. These unresolved issues collectively raise concerns about reproducibility, safety, and accountability.

Future adoption will require regulatory and technical frameworks that are both adaptive and transparent and that allow verification across institutions and populations. Data privacy laws, including the General Data Protection Regulation (GDPR), add additional complexity because they restrict cross-border data sharing and automated decision-making, which in turn shapes model development pipelines, data governance practices, and federated learning collaborations. International partnerships are needed to establish benchmark datasets that reflect global diversity, including representation from low- and middle-income settings. Clinicians should also be trained in hybrid human-AI collaboration within radiology curricula so that they can critically evaluate algorithmic outputs. Ethical and policy safeguards that balance fairness, patient rights, privacy protections, and commercial considerations are essential. Medico-legal issues similarly require close attention, particularly malpractice liability and insurance coverage for AI-assisted decisions. Radiologists may still be held legally responsible for diagnostic errors even when these errors stem from flawed algorithmic outputs, creating uncertainty around how responsibility should be shared among clinicians, developers, and healthcare institutions. Existing malpractice and indemnity frameworks were not designed for hybrid human-AI workflows, raising questions about coverage, compensation, and the standards of care expected when clinicians rely on AI recommendations. Addressing these medico-legal concerns will be critical for safe, accountable, and sustainable AI integration. Ultimately, the success of AI will depend on whether it evolves into an inclusive and adaptable ecosystem rather than a collection of isolated technological achievements.

## Conclusions

The application of AI to oncologic radiology is redefining the field, but much of the current literature has been piecemeal in either disease-specific applications or limited performance evaluations. In contrast to previous reviews that either catalog AI by cancer type or performance measures, this review presents a thematic synthesis, framing AI by cross-cutting frameworks that demonstrate how AI is redefining radiology as a systemic enabler of precision oncology. By prioritizing precision imaging, early detection, multimodal integration, explainability, federated learning, clinical decision support, prognosis, equity, and workforce transformation, it shows that the impact of AI extends beyond the technical innovation to impact systemic change throughout healthcare. Radiology is no longer limited to descriptive interpretation, but it is changing to become a predictive, collaborative, and ethically controlled field at the heart of precision oncology. The success of AI will not be measured only by its accuracy, but also by its transparency, inclusivity, and effectiveness in a variety of health systems. AI must complement the work of radiologists, not to be a substitute, as they need to be imaging, data, and clinical judgment integrators to enable longitudinal and patient-centered care. The most significant query that has arisen today is that of viewing AI expanding as an accessible and adaptable ecosystem. In order to do that, it will be required to realize international associations, open validation, and moral frameworks that emphasize equity and global accessibility. Only in this way will AI be able to achieve its potential of transforming cancer diagnosis at a global level. While AI continues to evolve, many clinical advances remain highly dependent on the foundations of radiomics and texture analysis. The widespread implementation of more advanced AI systems remains a future aspiration, contingent upon robust international standardization and large-scale validation.
